# A Retrospective Clinical Investigation of the Safety and Adverse Effects of Pantoprazole in Hospitalized Ruminants

**DOI:** 10.3389/fvets.2020.00097

**Published:** 2020-03-17

**Authors:** Joseph S. Smith, Austin R. Kosusnik, Jonathan P. Mochel

**Affiliations:** ^1^Veterinary Diagnostic and Production Animal Medicine, College of Veterinary Medicine, Iowa State University, Ames, IA, United States; ^2^Biomedical Sciences, College of Veterinary Medicine, Iowa State University, Ames, IA, United States; ^3^Systems Modelling and Reverse Translational Pharmacology, College of Veterinary Medicine, Iowa State University, Ames, IA, United States

**Keywords:** pantoprazole, ruminant, cattle, bovine, goats, caprine, sheep, ovine

## Abstract

Clinical safety data for the use of gastroprotectants in hospitalized ruminants is lacking. In human patients, multiple adverse effects are possible from the use of pantoprazole including hematologic and electrolyte abnormalities as well as anaphylaxis and edema. The medical records of all hospitalized cattle, goats, and sheep administered pantoprazole over an ~5-year period were retrospectively analyzed for adverse effects. Seventy-nine eligible patients were observed. Hypomagnesemia was observed after pantoprazole administration in 10 cattle; however, no significant changes were noted when compared to baseline before pantoprazole administration. Significant changes were noted in serum indicators of hepatic and renal function; however, these represented downward trends that were most likely clinically insignificant. Anaphylaxis after pantoprazole administration was not observed; however, seven cattle displayed edema after pantoprazole administration. Veterinary clinicians should be aware of the potential for hypomagnesemia in hospitalized ruminants being administered pantoprazole and monitor patients accordingly. While these preliminary retrospective results indicate that pantoprazole may be a safe adjunctive therapy in hospitalized ruminants, additional studies are necessary to further determine the safety and toxicity of pantoprazole in ruminants.

## Introduction

Proton pump inhibitors, such as pantoprazole, are benzimidazole drugs that target the final common pathway of acid production and, as such, are more potent than H2 receptor antagonists such as cimetidine. Pantoprazole functions as an irreversible proton pump inhibitor (PPI), which increases gastric pH via bonding with the hydrogen–potassium ATPase pump on the secretory surface of gastric parietal cells. It is routinely used in canine and feline practice, and its use has been described for foals (1). In alpacas (2), pantoprazole has been shown to increase third-compartment pH when given at a dose of 1 mg/kg intravenously or 2 mg/kg subcutaneously with high bioavailability when administered subcutaneously. Currently, no pharmacokinetic, pharmacodynamic, or safety studies for pantoprazole exist for cattle, sheep, or goats, although case reports of the use of pantoprazole in individual ruminant animals without observed adverse effects or toxicities do exist (3–6).

Treatments for abomasal ulceration in ruminants are limited. Coating agents, such as bismuth subsalicylate, exist, but no drug with active activity for gastroprotection is labeled for ruminant use. When presented with a lack of labeled options, food animal clinicians often have to consider using a drug in an off-label manner. A study with the H2 receptor antagonist famotidine exists for cattle. While that study demonstrates an increase in gastrointestinal pH, famotidine has to be administered intravenously and multiple times daily (7). Multiple daily intravenous administrations present a challenge for food animal treatment. A similar drug to pantoprazole, omeprazole, is available in oral formulations for equine use but does not have the bioavailability to be an effective food animal therapeutic, and intravenous formulations are limited. As a widely available and commonly used human drug, pantoprazole presents a potential candidate for gastroprotection in ruminants, and comparative data suggest that once-daily administration may be possible for conditions such as abomasal ulceration.

While no pharmacokinetic data for pantoprazole exist for cattle, sheep, or goats, data for some large animal species do exist, primarily alpacas and foals (1, 2). After intravenous administration to alpacas, a short elimination half-life (0.047 h) and high clearance (12.2 ml/kg/h) were noted for intravenous and subcutaneous administration (2). In that study's alpaca population, a bioavailability of 115% was observed after repeated subcutaneous dosing. Similarly, in foals, a short elimination half-life (1.43 h) and high clearance (80.6 ml/kg/h) were observed (1). Both of these studies also captured pharmacodynamic information, and in both species, a significant increase in gastric pH was noted after administration of pantoprazole. Currently, there are extremely limited options for gastroprotection in ruminant species. The efficacy of once daily intravenous (1.0 mg/kg) or subcutaneous (2.0 mg/kg) administration demonstrated in alpacas presents a compelling argument for clinicians to consider using this dosing protocol for other ruminant species that would need treatment with pantoprazole.

When used in humans for therapy, multiple adverse events have been reported from patients administered PPIs, mainly skin reactions, nephritis, pancytopenia, anaphylaxis, edema, hepatotoxicity, as well as biochemical changes such as hyponatremia and hypomagnesemia (8–14). Pantoprazole specifically has been associated with thrombocytopenia, hepatotoxicity, and pancreatitis (9, 11, 15–17). Currently, there are limited data on the toxicity and adverse effects of pantoprazole in veterinary species. The goal of this study was to investigate the clinical safety of pantoprazole used in ruminants for the treatment of gastrointestinal disease with respect to toxicity and adverse effects reported in other species.

## Methods

Medical records of the Food Animal and Camelid Hospital (FACH) of the Lloyd Veterinary Medical Center of Iowa State University's college of veterinary medicine were screened for visits of cattle, goats, and sheep that were administered 4 mg/ml pantoprazole sodium (pantoprazole sodium, Sun Pharmaceutical Industries, Inc.) from 01/01/2014 through 08/01/2019. Medical records were then scoured for patient information, the presence or absence of specific adverse effects associated with pantoprazole administration reported in humans, as well as complete blood counts (CBC) and serum biochemistry profiles collected up to 24 h prior to initiation of pantoprazole therapy and within 24 h post cessation of pantoprazole therapy. Focus was applied to electrolytes magnesium and potassium, as well as indicators of renal (blood urea nitrogen, creatinine) and liver function (AST, GGT). Hematology and chemistry analyzers were routinely calibrated and had daily quality controls performed per Iowa State University Clinical Pathology Laboratory protocol.

For serum magnesium and potassium levels, the values after pantoprazole administration were compared to normal species reference ranges.

Quantitative bloodwork values for magnesium, potassium, neutrophils, platelets, blood urea nitrogen (BUN), creatinine, AST, and GGT were calculated as:

Absolutedifference = Concentration(after pantoprazole administration) – Concentration (before pantoprazoleadministration).

Percent change of bloodwork values was calculated as:

Relative difference = Concentration (after pantoprazoleadministration) – Concentration (before pantoprazole administration)/(concentration before pantoprazoleadministration).

Due to the significant overlap of species parameter reference ranges as well as the small sample size (Table 1), the clinical pathology concentrations of animals with before and after values were pooled. Paired data were screened for normality via Shapiro-Wilk test and compared with the appropriate statistical test (paired *t* test for normally distributed data, and Wilcoxon test for nonparametric data) via a commercial software program (Prism 8.0.2, GraphPad Inc., La Jolla, CA). For all comparisons, a *P* < 0.05 was considered statistically significant.

**Table 1:** Demographic information of study population.

**Species**	**Total number**	**Male (*n*)**	**Female (*n*)**	**Age (years)**	**Breed (*n*)**
Bovine	43	24	19	0.6 ± 1.4	Mixed breed (14); Holstein (12); Aberdeen angus (7); Maine Anjou (3); Lincoln red shorthorn (2); Hereford (1); Miniature Hereford (1); Red Angus (1); Simmental (1); and Wagya (1)
Caprine	25	10	15	2.8 ±4.0	Boer (11); Mixed breed (9); La Mancha (2); Alpine (1); Nubian (1); and Nigerian dwarf (1)
Ovine	11	11	0	1.4 ± 1.0	Mixed breed (6); Hampshire Down (3); and Suffolk (2)

## Results

Forty-three cattle met the study inclusion criteria, 24 were male and 19 were female. Represented breeds were as follows: Mixed breed (*n* = 14); Holstein (*n* = 12), Aberdeen Angus (*n* = 7), Maine Anjou (*n* = 3), Lincoln red shorthorn (*n* = 2), Hereford (*n* = 1), Miniature Hereford (*n* = 1), Red Angus (*n* = 1), Simmental (*n* = 1), and Wagya (*n* = 1). Ages of study cattle were 0.60 ± 1.44 years. Twenty-five goats met inclusion criteria, 10 were male and 15 were female. Represented breeds were as follows: Boer (*n* = 11), Mixed breed (*n* = 9), La Mancha (*n* = 2), Alpine (*n* = 1), Nubian (*n* = 1), and Nigerian dwarf (*n* = 1). Ages of study goats were 2.76 ± 4.02 years. Eleven sheep met inclusion criteria, all were male. Represented breeds were as follows: Mixed breed (*n* = 6), Hampshire Down (*n* = 3), and Suffolk (*n* = 2). Ages of study sheep were 1.39 ± 1.04 years. All animals received additional therapies in the form of antimicrobials, anti-inflammatories, and/or intravenous fluids. Full demographic information is displayed in Table 1.

Thirty-six cattle received a 1.0 mg/kg dose of pantoprazole intravenously (I.V.) and seven cattle received a 2.0 mg/kg dose of pantoprazole subcutaneously (S.Q.). Fifteen goats received 1.0 mg/kg dose of pantoprazole I.V. and 10 goats received 2.0 mg/kg dose of pantoprazole S.Q. Seven sheep received 1.0 mg/kg dose of pantoprazole I.V. and four sheep received 2.0 mg/kg dose of pantoprazole S.Q. Study cattle, goats, and sheep received a total of (mean ± SD) 3.4 ± 2.3, 3.6 ± 1.6, and 5.9 ± 3.4 doses of pantoprazole during hospitalization, respectively.

**Figure 1:**
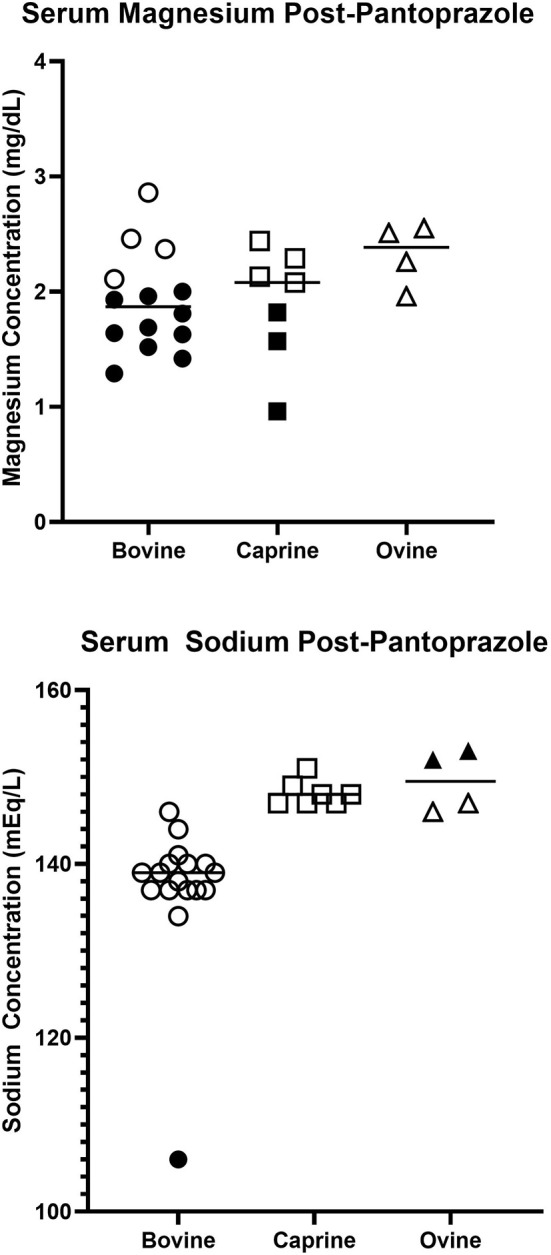
Serum magnesium **(Upper)** and sodium **(Lower)** concentrations in hospitalized bovine, caprine, and ovine patients after pantoprazole administration. Solid shapes indicate deviations from the normal reference range.

Fourteen cattle had serum magnesium measured after pantoprazole therapy. Ten of the 14 animals had magnesium below the normal reference range of {2.10–2.90} mg/dl. Seven goats had serum magnesium measured after administration of pantoprazole. Three goats had serum magnesium levels below the reference range of {1.85–2.6} mg/dl. Four sheep had post-pantoprazole serum magnesium levels measured, and none of these deviated from the reference range. Figure 1 displays the serum magnesium levels after pantoprazole administration for these animals.

Seventeen cattle had serum sodium levels measured after pantoprazole administration. One animal displayed sodium levels below the reference range 133–147 mEq/L. Seven goats had sodium levels measured after pantoprazole administration, and none of these values deviated from the reference ranges for goats. Four sheep had sodium measured, and two animals had serum sodium levels above the normal reference range. Figure 1 displays the serum sodium levels after pantoprazole administration for these animals.

Data for animals with data representative of hematologic and biochemistry values before and after pantoprazole administration are present in Table 2. Significant changes at the species level were observed for BUN in cattle (−30.8%; *P* = 0.0293), GGT in goats (−5.9%; *P* = 0.0367), as well as AST in cattle (−1.9%, *P* = 0.0059) and sheep (−23.8%; *P* = 0.0253). Moderate changes were observed for all other values at the species level, but none of these approached statistical significance (*P* < 0.05).

**Table 2:** Comparative reference ranges for various ruminant hematological and biochemical parameters as determined by the ISU Clinical Pathology Laboratory.

**Parameter**	**Bovine reference range**	**Caprine reference range**	**Ovine reference range**
Neutrophil (× 10^3^/μL)	0.6–4.0	1.2–7.2	0.7–6.0
Platelet (× 10^3^/μL)	100–800	300–600	250–800
Sodium (mEq/L)	133–147	140–151	136–150
Magnesium (mg/dL)	2.10–2.90	1.85–2.60	1.82–3.65
BUN (mg/dL)	7–32	19–34	14–25
Creatinine (mg/dL)	0.7–1.9	0.3 – 0.8	1.0–1.9
GGT (IU/L)	1–50	38–100	40–100
AST (IU/L)	68–156	60–160	55–150

When pooling data from multiple ruminant species, several parameters exhibited statistically significant differences before and after pantoprazole administration: neutrophil counts (*P* = 0.0400), BUN concentration (*P* = 0.0224), GGT concentration (*P* = 0.0011), and AST (*P* = 0.0150). No statistically significant differences were noted for concentrations of platelets (*P* = 0.6942), sodium (*P* = 0.7608), magnesium (*P* = 0.3039), or creatinine (*P* = 0.0665).

When concentrations were evaluated with respect to species, only 1 of 12 cattle were neutropenic and one out of three sheep were found to be thrombocytopenic. For biochemistry values, no animals were hyponatremic. One bovine and one out of three goats were azotemic. Seven of 10 cattle and two out of three goats were hypomagnesemic. Seven of 10 cattle and two out of three sheep had increased GGT after pantoprazole administration. Three out of nine cattle and two of four sheep had increased AST. Absolute and percentage difference of examined hematological and serum biochemistry values for all ruminants in this study can be viewed in Table 3.

**Table 3:** Absolute and percentage difference of examined hematological and serum biochemistry values by species of cattle, goats, and sheep administered pantoprazole.

**Parameter (unit)**	**Bovine difference (*n*)**	**Bovine difference %**	**Bovine *P***	**Caprine difference (*n*)**	**Caprine difference %**	**Caprine *P***	**Ovine difference (*n*)**	**Ovine difference %**	**Ovine *P***
Neutrophil (× 10^3^/μL)	0.84 ± 13.7 (11)	8.4	0.2061	−2.18 ± 6.03 (6)	0.9	0.4516	−4.05 ± 3.10 (3)	−53.4	0.1517
Platelet (× 10^3^/μL)	9 ± 264 (11)	12.3	0.9121	−98.5 ± 395.1 (6)	−9.2	0.5682	179 ± 359 (3)	49.7	0.4785
Sodium (mEq/L)	1.92 ± 5.25 (12)	1.5	0.2319	0 ± 2.64 (3)	0.02	0.999	−2.33 ± 4.73 (3)	−1.4	0.4825
Magnesium (mg/dL)	−0.078 ± 0.677 (10)	−0.4	0.7241	−0.24 ± 0.40 (3)	−15.3	0.4175	−1.19 ± 1.25 (3)	−28.7	0.241
BUN (mg/dL)	−9.75 ± 16.53 (12)	−30.8	0.0293	7.5 ± 13.7 (4)	39.2	0.3536	−71.7 ± 81.1 (3)	−60.0	0.2625
Creatinine (mg/dL)	−0.45 ± 0.81 (11)	−16.7	0.0752	−0.3 ± 0.6 (4)	26.9	0.5000	−3.17 ± 4.57 (3)	−47.5	0.3297
GGT (IU/L)	−112.3 ± 208.5 (10)	−26.5	0.1934	−3.5 ± 14.7 (3)	−5.9	0.0367	−34 ± 9.5 (3)	−23.0	0.2658
AST (IU/L)	−51.8 ± 107.5 (9)	−1.9	0.0059	−46.8 ± 25.95 (4)	−25.2	0.6657	−38.3 ± 43.4 (3)	−23.8	0.0253

*Difference described post-administration levels minus pre-administration baseline. P < 0.05 were considered statistically significant*.

No patients had suspected anaphylactic administrations after pantoprazole administration. Seven cattle demonstrated edema during the period in which they were administered pantoprazole. One 14-day-old calf displayed a neurologic episode immediately after pantoprazole administration. None of these adverse effects were noted in any of the small ruminants receiving pantoprazole.

## Discussion

There are currently no retrospective studies addressing the safety of use of pantoprazole in clinical cattle, goats, or sheep. The efficacy of pantoprazole has been reported and has been shown to increase pH of the third compartment in alpacas (2). However, that study did not evaluate safety with respect to adverse findings of pantoprazole administration in humans. Case reports involving ruminant species demonstrate use of pantoprazole in goats (3, 18), a sheep (19), a beef bull (6), a camel (4), and a yak (5) with no reported complications from administration. Our study provides clinically relevant data to guide veterinary practitioners for the use of pantoprazole in hospitalized ruminants.

In people, thrombocytopenia has been reported after PPI administration. Omeprazole, pantoprazole, and lansoprazole all have reported cases of thrombocytopenia after administration (15, 20, 21). These cases of thrombocytopenia are thought to be due to long-term use (22). This mechanism is thought to be mediated by a reduced expression of adhesive and inflammatory proteins due to the reactivity toward hydroxyl radicals that pantoprazole possesses (23). This mechanism is also hypothesized to be a cause of acute pancreatitis in rats experimentally administered pantoprazole (23). No significant differences in platelet concentrations were noted in the patient population of this study.

Hypomagnesemia is an incompletely understood adverse effect of PPI usage in people. It is hypothesized in people that long-term usage and patient age (elderly individuals more likely effected) may influence PPI-induced hypomagnesemia (9). While several animals had serum magnesium levels below the reference ranges after pantoprazole administration, no significant differences were seen in magnesium levels in the patient population administered pantoprazole in this study when compared to baseline levels before pantoprazole administration.

Hyponatremia is a reported risk in human populations administered PPI with cases suggesting that age may play a role (12). In our study, hyponatremia was only observed in one animal after pantoprazole administration, and no significant differences were noted in the animals that had sodium levels measured before and after pantoprazole administration.

Hepatotoxicity has been reported in the use of PPI in people. In one case, the liver function improved 1 week after discontinuation of oral pantoprazole therapy and the patient made a full recovery (8). While significant differences were noted before and after administration of pantoprazole in our study in liver enzymes, primarily GGT, this does not likely suggest hepatotoxicity as the changes suggested decreased enzyme concentrations after pantoprazole administration.

Anaphylaxis has been reported in people after administration of pantoprazole tablets, manifesting as edema, pruritus, nausea, vomiting, respiratory distress, and rashes (13). In people, anaphylactic reactions have been observed with omeprazole, pantoprazole, and lansoprazole skin prick tests by one study, and the same study suggests that cross-reactivity may exist in people to proton pump inhibitors (14). While none of the patients in our study demonstrated anaphylaxis, clinicians should be aware that this might be a possibility based on the comparative literature.

Edema has been reported as an adverse effect in people administered pantoprazole. Peripheral edema has been noted in female patients administered pantoprazole, omeprazole, or lansoprazole at standard dosages, resolving 2–3 days after drug discontinuation (24). Due to the gender predilection, a competitive inhibition of the receptor of water regulation hormones was suspected. Of the seven cattle that displayed edema during pantoprazole therapy, four were female.

While all biochemistry and hematology values presented changes before and after pantoprazole administration at the species level, the majority were not clinically significant (*P* < 0.05). Of the values that displayed statistically significant changes (BUN in cattle, GGT in goats, and AST in cattle and sheep), the clinical significance is likely marginal as all of these values decreased after pantoprazole administration, and these values typically increase in instances of organ dysfunction as would be encountered with an adverse drug reaction. Similarly for the pooled changes across species, changes were noted in neutrophil counts, BUN, GGT, and AST that reached statistical significance, but also demonstrated downward trends after pantoprazole administration. While this is encouraging for clinical safety, this should also reaffirm that clinicians monitoring food animals receiving pantoprazole should observe these values for changes.

This study had several limitations. These include the small patient population as well as the retrospective nature of the analysis. This is particularly relevant as much variation was noted in patient breed, age, and presenting etiology. As such, future prospective (placebo-controlled) studies are needed to confirm the safety of pantoprazole use in cattle, goats, and sheep. Similarly, as this work is to serve as an initial exploration of the safety of pantoprazole in hospitalized ruminants, additional prospective studies will be necessary to further determine the clinical safety. While a limitation exists in the use of a patient population that is administered other medications, our study population provides a benefit in that these are actual patients treated for clinical disease, and disease has been noted to alter actions of pharmacokinetics and pharmacodynamics in ruminants. Additional studies will be necessary to identify toxicity, as well as off-target or epigenetic effects that pantoprazole could may have in ruminant species, as these mechanisms could provide insight into adverse effect potential in this patient population (25). As all of the animals in this study were presented to a teaching hospital for disease, there is a strong possibility that concurrent treatment, stage of disease process, differences in drug exposure, and other factors could have influenced the measured variables of this work. Of importance, for all patients in this study, pantoprazole was used in an extralabel fashion. Clinicians should be aware that there are limited data available for advising clients on withdraw times for pantoprazole in food animal species at this time, so consultation with the appropriate organization, such as gFARAD, should be considered. Practitioners should be aware that due to regulatory differences, extralabel use of pantoprazole in ruminant food animal species may not be permissible in all countries.

## Conclusions

Our study's relevant clinical findings indicate that pantoprazole may be a safe gastroprotectant therapy for hospitalized ruminants. Clinically significant hyponatremia, thrombocytopenia, hepatic enzyme changes, evidence of nephritis, or anaphylaxis was not observed. Hypomagnesemia was observed in some cases, but may be more reflective of systemic disease than pantoprazole administration as no significant changes were noted in serum magnesium concentration in animals that had concentrations measured before and after pantoprazole administration. Edema was observed in one patient. Additional studies are necessary to elucidate the complete clinical safety of pantoprazole in ruminants; however, our preliminary investigation provides support that pantoprazole may be a safe adjunctive gastroprotectant therapy in hospitalized ruminants.

## Data Availability Statement

The datasets generated for this study are available on request to the corresponding author.

## Ethics Statement

Ethical approval was not required for this study according to national/local legislation because this was a retrospective study that evaluated the medical records of these patients. No animal research was done, other than scouring the hospitalization records.

## Author Contributions

JS managed clinical cases, aided in study design and data collection, analyzed data, and composed the manuscript. AK aided in case management, data collection, and analysis. JM aided in study design, data analysis, and manuscript construction. All authors approve of this manuscript.

### Conflict of Interest

The authors declare that the research was conducted in the absence of any commercial or financial relationships that could be construed as a potential conflict of interest.
